# Low magnitude vibration alleviates age-related bone loss by inhibiting cell senescence of osteogenic cells in naturally senescent rats

**DOI:** 10.18632/aging.202907

**Published:** 2021-04-22

**Authors:** Jirui Wen, Mingyue Bao, Min Tang, Xueling He, Xinghong Yao, Liang Li

**Affiliations:** 1 Institute of Biomedical Engineering, West China School of Basic Medical Science and Forensic Medicine, Sichuan University, Chengdu 610041, Sichuan, China; 2 Laboratory Animal Center, Sichuan University, Chengdu 610041, Sichuan, China

**Keywords:** osteoporosis, senescence, low magnitude vibration, osteogenesis

## Abstract

Dysfunction of bone marrow mesenchymal stem cells (BMSCs), osteoblasts and osteocytes may be one of the main causes of bone loss in the elderly. In the present study, we found osteogenic cells from aged rats all exhibited senescence changes, with the most pronounced senescence changes in osteocytes. Meanwhile, the proliferative capacity and functional activity of osteogenic cells from aged rats were suppressed. Osteogenic differentiation capacity of BMSCs from aged rats decreased while adipogenic capacity increased. The mineralization capacity, ALP activity and osteogenic proteins expression of osteoblasts from aged rats decreased. Additionally, osteocytes from aged rats up-expressed sclerosteosis protein, a negative regulator of bone formation. To inhibit osteogenic cell senescence, we use low magnitude vibration (LMV) to eliminate the senescent osteogenic cells. After LMV treatment, the number of osteogenic cells staining positively for senescence-associated-β-galactosidase (SA-β-Gal) decreased significantly. Besides, the expression of anti-aging protein SIRT1 was upregulated significantly, while p53 and p21 were downregulated significantly after LMV treatment. Thus, the LMV can inhibit the senescence of osteogenic cells partly through the Sirt1/p53/p21 axis. Furthermore, LMV was found to promote bone formation of aged rats. These results suggest that the inhibition of osteogenic cell senescence by LMV is a valuable treatment to prevent or delay osteoporosis.

## INTRODUCTION

Osteoporosis is a type of systemic bone disease characterized by decreased bone density, increased bone fragility, bone microstructure destruction, and increased risk of fractures [1]. Aging is a risk factor for many chronic diseases including osteoporosis. In recent years, many studies have revealed the bone changes that occur with age from both genetic and environmental perspectives [2]. Therefore, there is an urgent need to prevent or reverse age-related bone loss so as to maximize the healthy lifespan of humans.

Cell senescence refers to the process of irreversible cell cycle arrest under various damage stimuli [3]. With age, senescent cells gather in a variety of tissues, forming their unique phenotypic characteristics. Most of the senescent cells become larger and flatter, and have a higher β-galactosidase activity [4]. In addition, cyclin-dependent kinase inhibitor 2A (CDKN2A) and cyclin-dependent kinase inhibitor 2B (CDKN2B) are transcriptionally activated in senescent cells, and their gene products p16^INK4a^ and p21 are considered to be aging effectors, causing cell cycle arrest and cell proliferation delay [5, 6]. At the same time, due to nuclear DNA damage and mitochondrial dysfunction of senescent cells, the chromatin of senescent cells will undergo significant changes, manifested as permanent DNA damage and obvious centromeric satellite heterochromatin depolymerization [7].

As a potential target for the prevention and treatment of a variety of senile diseases, the mechanism of cellular senescence is a common potential risk factor for a variety of chronic diseases of the elderly. Studies have shown that there is a close connection between the accumulation of senescent cells and aging diseases, and the accumulation of senescent cells will cause a variety of aging phenotypes and pathological changes [8, 9]. Studies have found that the administration of the drug senolytics to induce the elimination of senescent cells in mice can delay the occurrence of aging changes in various tissues such as adipose tissue, skeletal muscle and eyes, and significantly increase the life span of the mice [10]. Cell senescence is also closely related to the pathological changes of bone tissue. Farr et al. studied the effect of eliminating senescent cells on age-related bone loss by establishing a mouse model of osteoporosis. They found that senescent cells are associated with age-related bone loss. And they proved that senescent cells as a therapeutic target can inhibit bone resorption and promote bone formation [11]. Farr et al. also identified various cell types in the bone microenvironment, and the results showed that cells of different lineages in the bone microenvironment, including osteoblast progenitor cells, osteoblasts, osteocytes and myeloid cells, both will gradually become senescent. But senescence-associated secretory phenotype is mainly produced in osteocytes and myeloid cells [7]. These accumulated senescent cells may be an important reason for accelerating bone loss. Notably, although both rats and mice have been used extensively to characterize age-related changes in bone [12], as far as we know, the senescent osteogenic cells in rat model have not been identified. Whether the osteogenic cells in rats have similar senescence changes as the mice do is a key question. By identification of senescent osteogenic cells in rat model, we can more clearly analyze the cytological changes in the bone microenvironment of aged rats, so as to provide help for the better application of rat model in the basic research of senile osteoporosis.

As a non-drug, non-invasive systemic exercise therapy, low magnitude vibration (LMV) with acceleration less than 1 g (g = 9.81 m/s^2^) had been proved to promote bone formation [13]. Studies had also reported that vibration with the acceleration <1 g and frequency 20-90 Hz can increase bone volume and bone density of humans and animal models [14–16]. However, whether low magnitude vibration can regulate the osteogenic cells in the aged rats remains unclear. Is it a potential anti-aging therapy and can it improve the bone microenvironment in the elderly? The targeted elimination of senescent osteogenic cells by low magnitude vibration needs further verification.

Although Farr et al. did a pioneering and outstanding work to identify the aging changes of various cell types in the bone microenvironment, the impact of aging on the biological functions of osteogenic cells is not fully understood, and which cell type in the bone microenvironment has the most obvious changes with age is also little known. In addition, whether low magnitude vibration can eliminate the osteogenic cells in the aged rats remains unclear. Therefore, this study compared the differences in aging changes that occur in various osteogenic cells, studied the impact of cell senescence on the biological functions of various osteogenic cells, and explored the potential scavenging effect of low magnitude vibration on senescent osteogenic cells.

## MATERIALS AND METHODS

### Animals

3-month-old male (n=10) and female (n=20) SD rats were selected as the control group and 22-month-old male (n=10) and female (n=30) SD rats were selected as the elderly group. All rats were healthy specific pathogen free (SPF) animals and were purchased from the Experimental Animal Center of Sichuan University. All rats were exposed to a relative humidity of 40%-70% and a temperature of 18-26° C. Animal experiments were approved by ethics committee of Sichuan University.

### Isolation, culture and identification of BMSCs, osteoblasts and osteocytes

BMSCs were isolated as References [17] reported, simply described, the bone marrow was flushed out with serum-free medium using a 10 mL syringe, collected in a centrifuge tube at 800 rpm for 5 min, and the supernatant was discarded to retain the cell precipitate. Then cells were incubated at 37° C in 5% CO2 incubator; after 3 hours, the first change of culture medium was made; then the culture medium was changed every three days. Adherent cells were passaged at 80%-90% confluence and the passages 3-4 were used for the subsequent experiments. The surface markers of BMSCs were analyzed by flow cytometry assay to identify BMSCs.

Osteoblasts were isolated as References [18] reported, simply described, the femur and tibia were separated aseptically, and the bone fragments were cut into 0.1 cm × 0.1 cm pieces using bone gnashing forceps, then the bone fragments were sequentially digested by collagenase I at 37° C. The digestion solution was collected, centrifuged and resuspended in a culture flask. The medium was changed every 3 days. The osteoblasts were identified by alizarin red staining and alkaline phosphatase (ALP) staining of P3-P4 generation osteoblast from 3-month-old female SD rats.

Osteocytes were isolated as References [19] reported, simply described, the femur and tibia were separated aseptically into 0.1 cm × 0.1 cm bone fragments, and the bone fragments were digested sequentially at 37° C with collagenase I and EDTA. When the fusion degree of primary osteoblasts reached 80-90%, the osteoblasts were digested and passed to the next generation. The osteocytes were identified by E11/gp38 immunofluorescence staining of P3-P4 generation osteocytes from 3-month-old female SD rats.

### Senescence-associated heterochromatic foci (SAHF) and aging-related DNA damage detection

Cells were seeded on coverslip and fixed with 4% paraformaldehyde for 15 min, then permeabilized in 0.1% Triton X-100 for 15 min and blocked with 2% bovine serum albumin for 30 min. Primary antibodies anti-H3K9me3 (CST, USA) or 53BP1 (Abcam, USA) were added at room temperature for 1 h. Then fluorescent secondary antibodies were added for 2 h at room temperature. Next, DAPI was used to counterstain nuclei. SAHF formation was detected by observation of the fluorescence signals of H3K9me3, and fluorescence signals of 53BP1 was observed to detect DNA damage.

### SA-β-gal staining

Cells were seeded on coverslip and fixed with 4% paraformaldehyde for 15 min, and rinsed twice with PBS. Then the cells were incubated with SA-β-gal reaction solution (Beyotime, China) at 37° C for 12 h, rinsed twice with double-steaming water, observed and counted under the optical microscope.

### CCK-8 detection

Cells from each group were seeded on 96-well plates at a density of 3×10^3^/well. 10 μL CCK-8 reaction solution (ApexBio, USA) was added to each well after indicated times (1d, 3d, 5d and 7d) of natural growth, and incubated for 2 h. The absorbance was measured at 450 nm using a microplate reader.

### Alkaline phosphatase staining

The cells were fixed with 95% ethanol for 10 min, stained with alkaline phosphatase staining solution (Beyotime, China) for 20 min, rinsed three times with ddH2O, observed and photographed under the optical microscope.

### Alizarin red staining

Cells were fixed with 95% ethanol for 10 min, stained with 0.2% alizarin red staining solution (Solarbio, China) for 30 min, rinsed three times with ddH2O, observed and photographed under the optical microscope.

### Detection of alkaline phosphatase activity

Alkaline phosphatase activity was detected by alkaline phosphatase (ALP) assay kit (Beyotime, China) following the instructions of the manufacturer. Briefly, 50 μL of samples or standard reagents were incubated with a chromogenic substrate for 30 minutes at 37° C, and the OD value in each group was detected at 405 nm.

### Oil red o staining

The cells were fixed with 95% ethanol for 10 min, and 0.3% Oil Red O staining solution (Solarbio, China) was added for 15 min. Then the cells were rinsed with ddH2O three times, and the lipid droplets were observed and photographed under the optical microscope.

### Western blot

The proteins were lysed and collected by cell lysis solution, and the concentration of proteins was detected by BCA kit. Total protein was separated by SDS-PAGE electrophoresis and then transferred onto 0.22 μm PVDF membranes. After that, membranes were blocked and then incubated with primary antibodies overnight at 4° C. After incubated with a secondary antibody for 1 hour at room temperature, the blots were visualized using an ECL kit (Pierce, USA) and quantified with Image J software.

### Application of LMV to osteogenic cells and rats

To analyze the effect of LMV on osteogenic cells, the osteogenic cells from old female rats were divided into static group and vibration groups (cells were vibrated at 0.3 g and 90 Hz). For the vibration groups, osteogenic cells were seeded in six-well plates, then the plates were fixed in a box and placed on the vibration platform of the vibration device (ZD-50T, Guangzhou Meiyifeng Test Equipment CO., Ltd, China), and vibrated for 30 minutes, 5 days. To test the effect of vibration at 0.3 g and 90 Hz on the bones of aged rats, 20 of 22-month-old female rats were randomly divided into the static group (n = 10) and vibration group (n = 10). For rats, a vertical whole-body vibration at 0.3 g and 90 Hz was performed for 30 minutes, once daily, 5 days a week until 12 weeks for subsequent analysis.

### μCT

The left femur was dissected from the rats, then placed into the scanner Viva CT 80 for scanning. The scanning parameters were as follows: a voltage of 70 kV and a current of 114 μA with a resolution of 12 μm per pixel. The images were analyzed with the μCT Tomography software, and the region of interest (ROI) selected for analysis was from 1.5 to 4.5 mm below the growth plate to determine trabecular bone volume per tissue volume (Tb. BV/TV), trabecular number (Tb. N) and trabecular separation (Tb. Sp).

### Morphological staining of bone tissue

The bone tissue section of the proximal femur was selected, and the morphology of bone tissue was observed by HE staining and Masson staining, and the percentage of trabecular bone area was analyzed by Image J software.

### Analysis of serum biomarkers

Alkaline phosphatase activity and tartrate acid phosphatase activity was detected by alkaline phosphatase assay kit and tartaric acid-resistant acid phosphatase assay kit following the instructions of the manufacturer (Beyotime, China).

### Statistical analysis

Replicated animals were used as replication. Data were analyzed by SPSS 20.0 software and presented as mean ± SD. Comparisons were analyzed using Student's t test for experiments with two groups, or one-way ANOVA for multiple groups. Differences were considered significant at P < 0.05.

## RESULTS

### Isolation and identification of osteogenic cells from young rats

**Figure 1:**
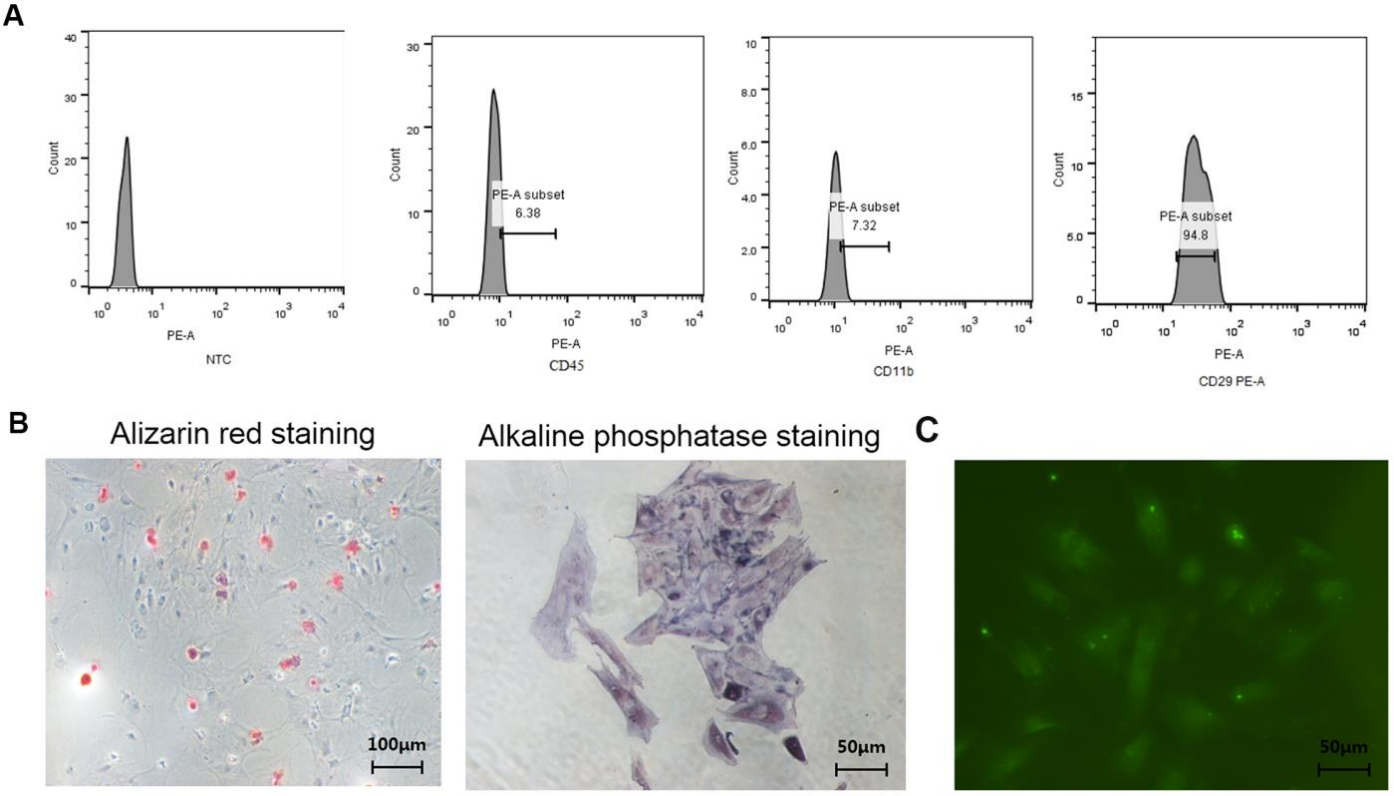
**Identification of BMSCs, osteoblasts, and osteocytes from normal female rats.** (**A**) Identification of surface antigens in BMSCs from normal female rats; (**B**) Alizarin red staining and ALP staining of osteoblasts from normal female rats; (**C**) E11/gp38 immunofluorescence staining of osteocytes from normal female rats.

As shown in Figure 1A, the CD29 positive rate of isolated BMSCs reached 94.8%, the CD45 negative rate reached 93.62%, and the CD11b negative rate reached 92.68%, indicating the purity of the isolated BMSCs. As shown in Figure 1B, the osteoblasts isolated and cultured were positive for Alizarin Red staining and the ALP staining, indicating the purity of the osteoblasts. As shown in Figure 1C, the isolated osteocytes highly expressed E11/gp38, indicating the purity of the osteocytes.

### Senescence detection of BMSCs in aged rats and young rats

**Figure 2:**
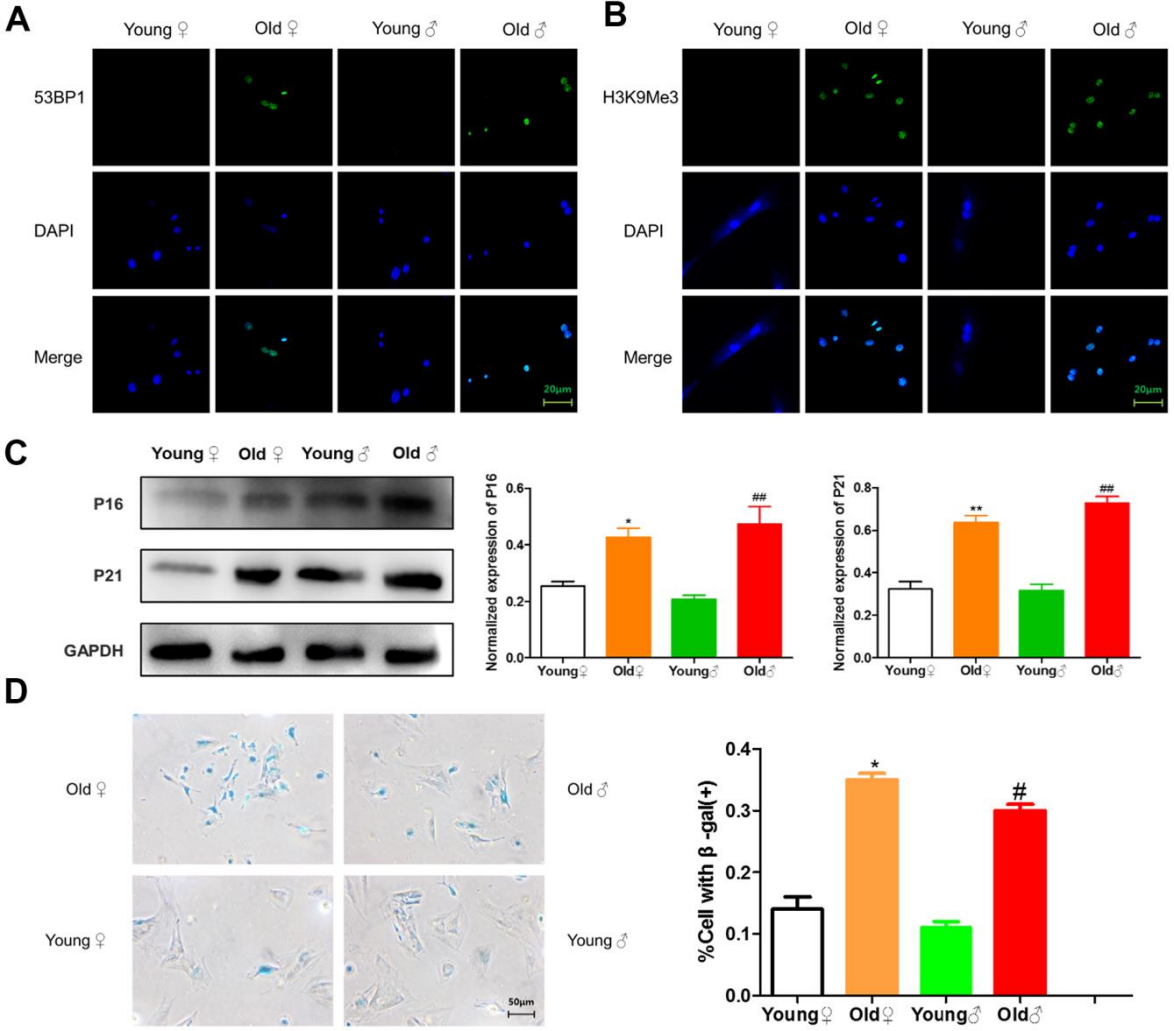
**Senescence identification of BMSCs in aged and young rats.** (**A**) 53BP1 immunofluorescence staining of aged and young rat BMSCs; (**B**) H3K9Me3 immunofluorescence staining of aged and young rat BMSCs; (**C**) Senescence marker protein assay and relative protein expression statistics of aged and young rat BMSCs $\left(\bar{x}\pm s,\text{n}=3\right)$; (**D**) β-Galactosidase staining plots and statistical plots of the percentage of positive β-galactosidase staining cells $\left(\bar{x}\pm s,\text{n}=3\right)$; *P < 0.05 vs. young female rats, **P < 0.01 vs. young female rats, ^#^P < 0.05 vs. young male rats, ^##^P < 0.01 vs. young male rats.

As shown in Figure 2A, 2B, green immunofluorescence showed the higher expression levels of 53BP1 and H3K9Me3 in aged rat BMSCs, indicating that aged rat BMSCs had DNA damage and senescence-related heterochromatin aggregation. As shown in Figure 2C, BMSCs from aged rats highly expressed senescence-related marker proteins p16^INK4a^ and p21. As shown in Figure 2D, the cells stained blue were β-galactosidase staining positive cells, and the β-galactosidase staining-positive BMSCs in aged rats were significantly higher than those in young rats.

### Senescence detection of osteoblasts in aged rats and young rats

**Figure 3:**
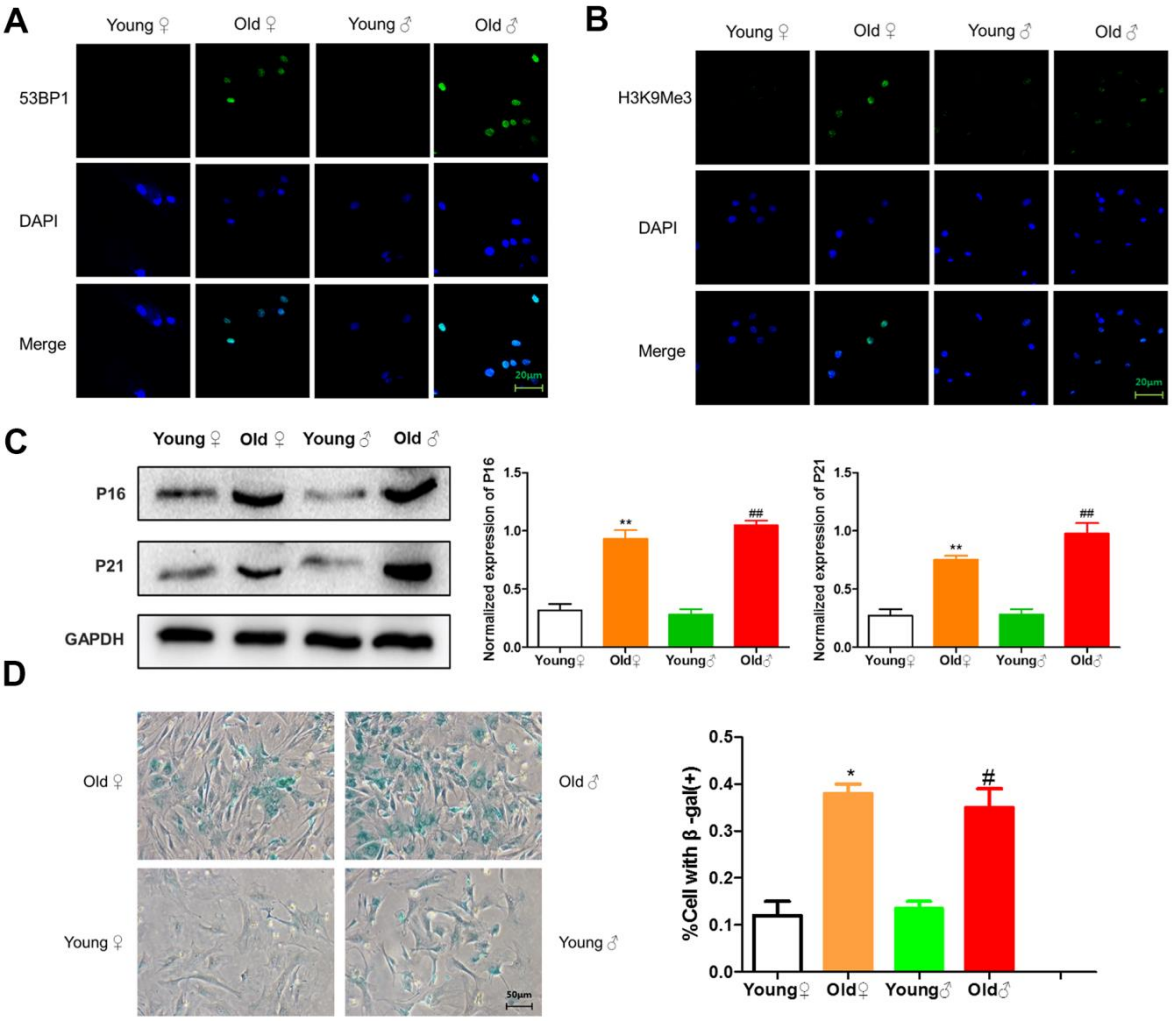
**Senescence identification of osteoblasts in aged and young rats.** (**A**) 53BP1 immunofluorescence staining of aged rat and young rat osteoblasts; (**B**) H3K9Me3 immunofluorescence staining of aged rat and young rat osteoblasts; (**C**) Senescence marker protein assay and protein relative expression statistics of aged rat and young rat osteoblasts $\left(\bar{x}\pm s,\text{n}=3\right)$; (**D**) β-Galactosidase staining plots and statistical plots of the percentage of positive β-galactosidase staining cells $\left(\bar{x}\pm s,\text{n}=3\right)$; *P < 0.05 vs. young female rats, **P < 0.01 vs. young female rats, ^#^P < 0.05 vs. young male rats, ^##^P < 0.01 vs. young male rats.

As shown in Figure 3A, 3B, green immunofluorescence showed the higher expression levels of 53BP1 and H3K9Me3 in aged rat osteoblasts, indicating that aged rat osteoblasts had DNA damage and senescence-related heterochromatin aggregation. As shown in Figure 3C, osteoblasts from aged rats highly expressed senescence-related marker proteins p16^INK4a^ and p21. As shown in Figure 3D, the β-galactosidase staining-positive osteoblasts in aged rats were significantly higher than those in young rats.

### Senescence detection of osteocytes in aged rats and young rats

**Figure 4:**
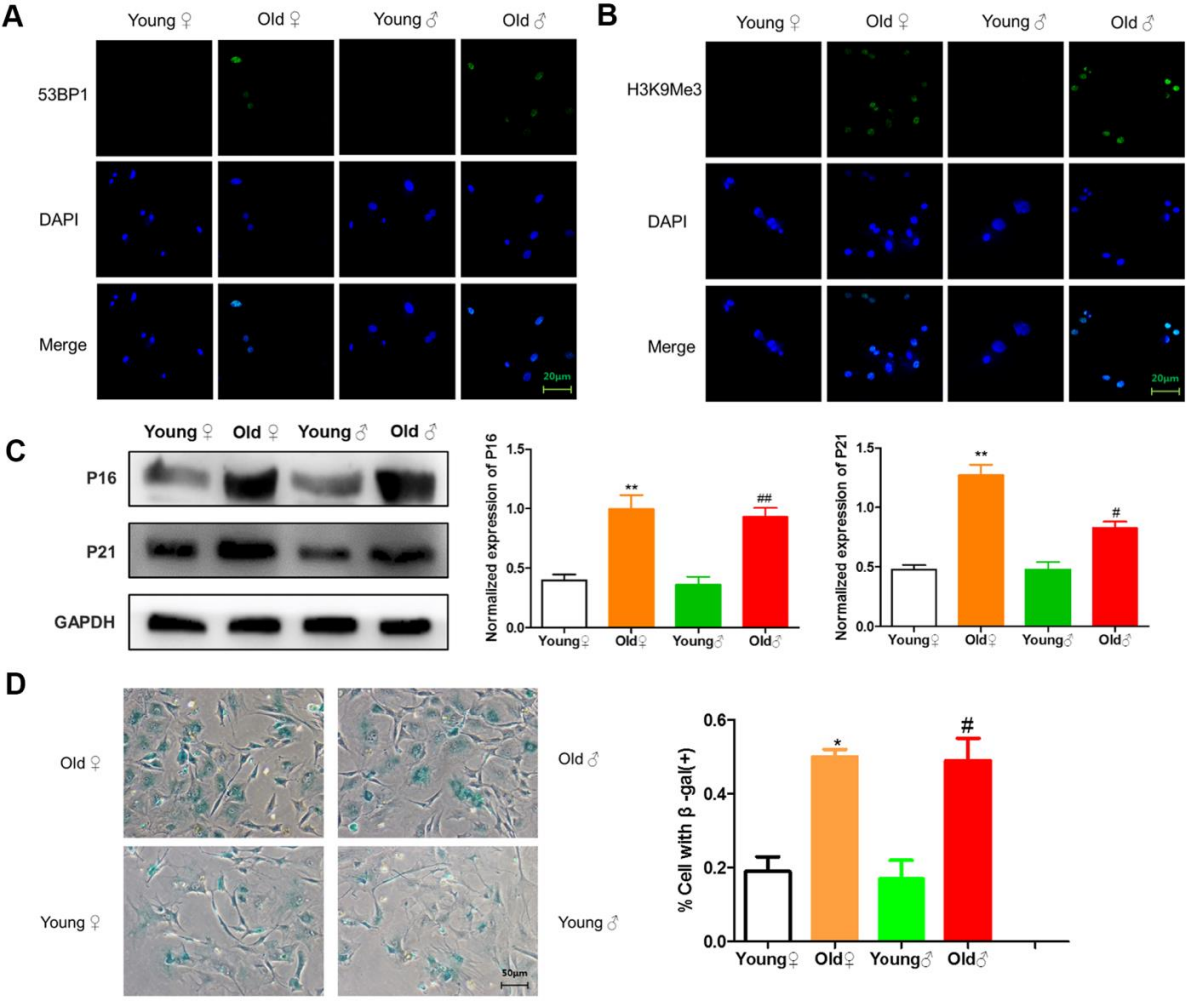
**Senescence identification of osteocytes in aged and young rats.** (**A**) 53BP1 immunofluorescence staining of aged rat and young rat osteocytes; (**B**) H3K9Me3 immunofluorescence staining of aged rat and young rat osteocytes; (**C**) Senescence marker protein assay and protein relative expression statistics of aged rat and young rat osteocytes $\left(\bar{x}\pm s,\text{n}=3\right)$; (**D**) β-Galactosidase staining of aged rat and young rat osteocytes and statistical plots of the percentage of positive β-galactosidase staining cells $\left(\bar{x}\pm s,\text{n}=3\right)$; *P < 0.05 vs. young female rats, **P < 0.01 vs. young female rats, ^#^P < 0.05 vs. young male rats, ^##^P < 0.01 vs. young male rats.

As shown in Figure 4A, 4B, 53BP1 and H3K9Me3 with green fluorescence were expressed in the nucleus of aged rat osteocytes, indicating that aged rat osteocytes had DNA damage and senescence-related heterochromatin aggregation. As shown in Figure 4C, aged rat osteocytes expressed senescence-related marker proteins p16^INK4a^ and p21. As shown in Figure 4D, the β-galactosidase staining-positive osteocytes in aged rats were significantly higher than those in young rats.

### Comparison of the proliferation activity of osteogenic cells between aged rats and young rats

**Figure 5:**
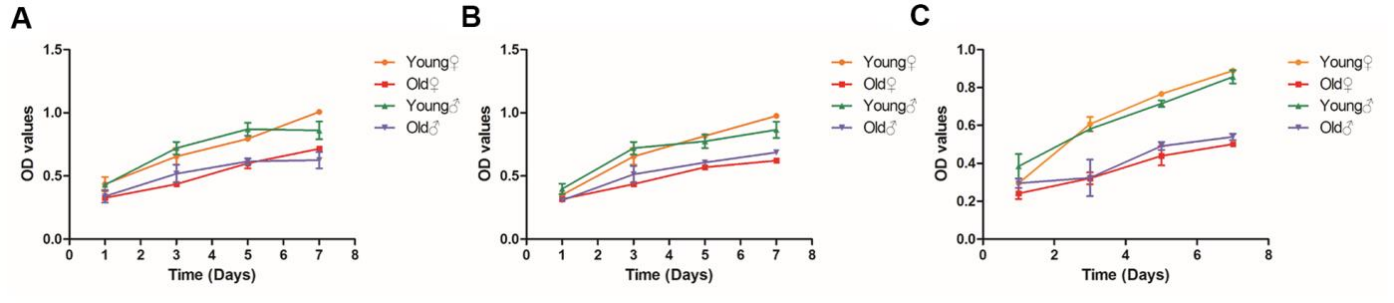
**Growth curves of BMSCs, osteoblasts and osteocytes in aged rats and young rats.** (**A**) Growth curves of BMSCs in aged rats and young rats; (**B**) Growth curves of osteoblasts in aged rats and young rats; (**C**) Growth curves of osteocytes in aged rats and young rats.

As shown in Figure 5, the OD values of BMSCs, osteoblasts and osteocytes of aged rats were significantly lower than those of young rats at each time point after culture, and the proliferation curves of BMSCs, osteoblasts and osteocytes of aged rats were lower than those of young rats, indicating that the proliferation ability of BMSCs, osteoblasts and osteocytes of aged rats was significantly decreased. It is noteworthy that at 7 days after inoculation, the OD value of osteocytes from 22-month-old female rats decreased by 43.6% compared with those of 3-month-old female rats and the OD value of osteocytes from 22-month-old male rats decreased by 37.1% compared with those from 3-month-old male rats, indicating osteocytes had the most obvious decrease in proliferation.

### Comparison of the functional activity of osteogenic cells between aged rats and young rats

**Figure 6:**
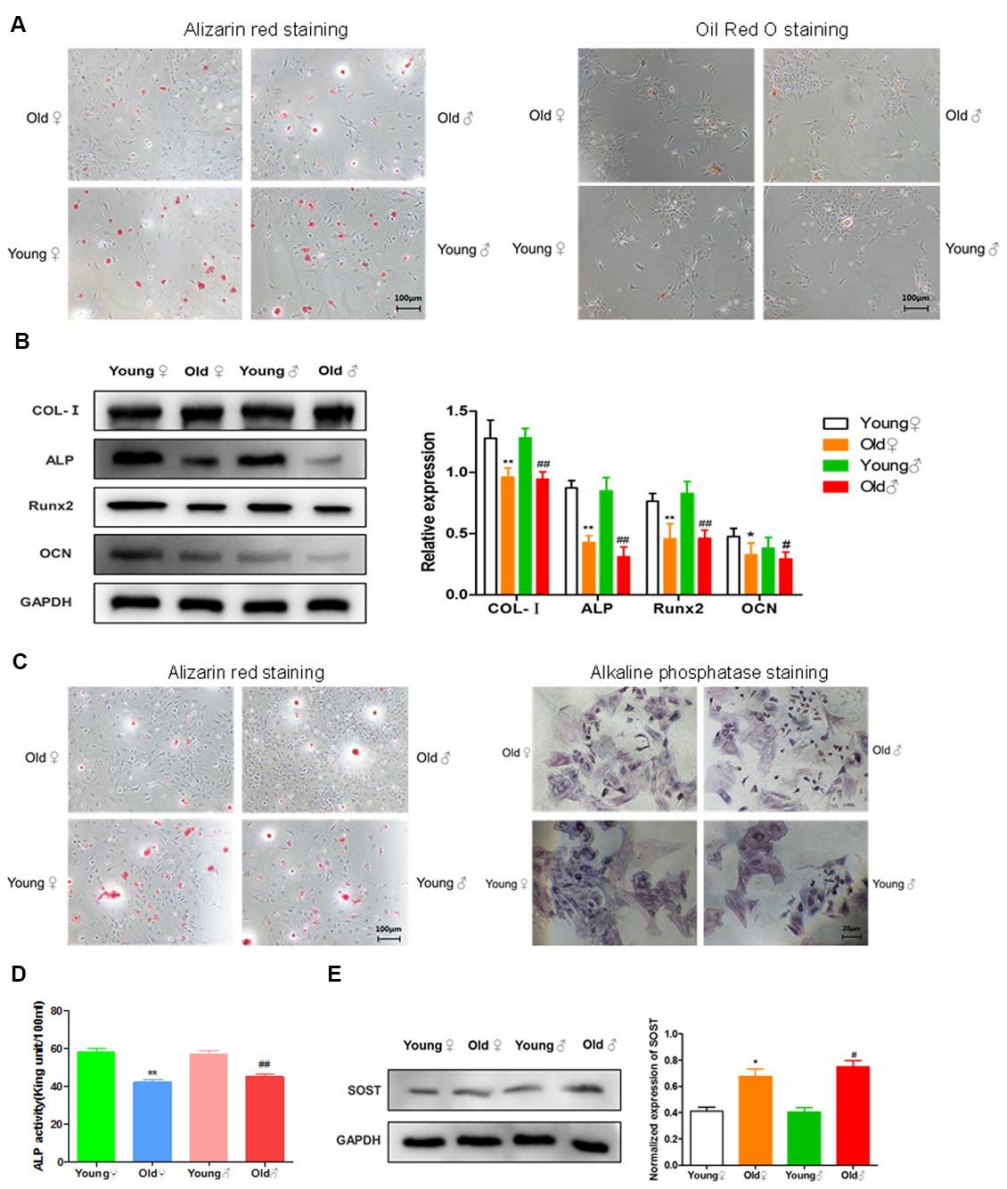
**Functional activities of BMSCs, osteoblasts and osteocytes in aged rats compared to young rats.** (**A**) Comparison of osteogenic and adipogenic differentiation ability of BMSCs between aged rats and young rats. BMSCs were cultured in osteogenic induction medium for 21 days, and alizarin red staining was performed. BMSCs were stained with oil red O after 14 days of culture in adipogenic induction medium, and the red drops in the figure were lipid droplets; (**B**) Determination of bone formation protein and statistics of protein relative expression in osteoblasts of aged rats and young rats $\left(\bar{x}\pm s,\text{n}=3\right)$; (**C**) Alizarin red staining and alkaline phosphatase staining of osteoblasts from aged rats and young rats; (**D**) The activity of alkaline phosphatase (ALP) in osteoblasts from old rats and young rats was measured $\left(\bar{x}\pm s,\text{n}=6\right)$; (**E**) The determination of sclerosteosis and the statistics of relative protein expression in osteocytes from old rats and young rats $\left(\bar{x}\pm s,\text{n}=3\right)$; *P < 0.05 vs. young female rats, **P < 0.01 vs. young female rats, ^#^P < 0.05 vs. young male rats, ^##^P < 0.01 vs. young male rats.

As shown in Figure 6A, after osteogenic induced differentiation, the calcium nodule formation was significantly reduced in aged rat BMSCs compared with young rat BMSCs. However, after adipogenic induced differentiation, the lipid droplet formation was significantly more in aged rat BMSCs than in young rat BMSCs, indicating that the adipogenic differentiation potential of aged rat BMSCs was higher than osteogenic differentiation potential. Western blot was used to detect the expression of COL-I, ALP, Runx2, OCN in the osteoblasts of elderly rats and young rats. As shown in Figure 6B, the expression of osteogenic proteins in the osteoblasts from aged rats was significantly decreased compared with those of young rats. In addition, as shown in Figure 6C, 6D, the number of calcified nodules, the staining depth of alkaline phosphatase staining, and the activity of alkaline phosphatase in the osteoblasts from aged rats were all lower than those in young rats, indicating that the osteogenic activity of aged rat osteoblasts was reduced. Western blot was used to detect the expression of sclerosteosis protein in osteocytes of elderly rats and young rats. As shown in Figure 6E, the osteocytes from aged rats highly expressed sclerosteosis, a negative regulator of bone formation, indicating the functional activity of aged rat osteocytes was significantly lower than that of young rats.

### Low magnitude vibration inhibited osteogenic cells senescence

**Figure 7:**
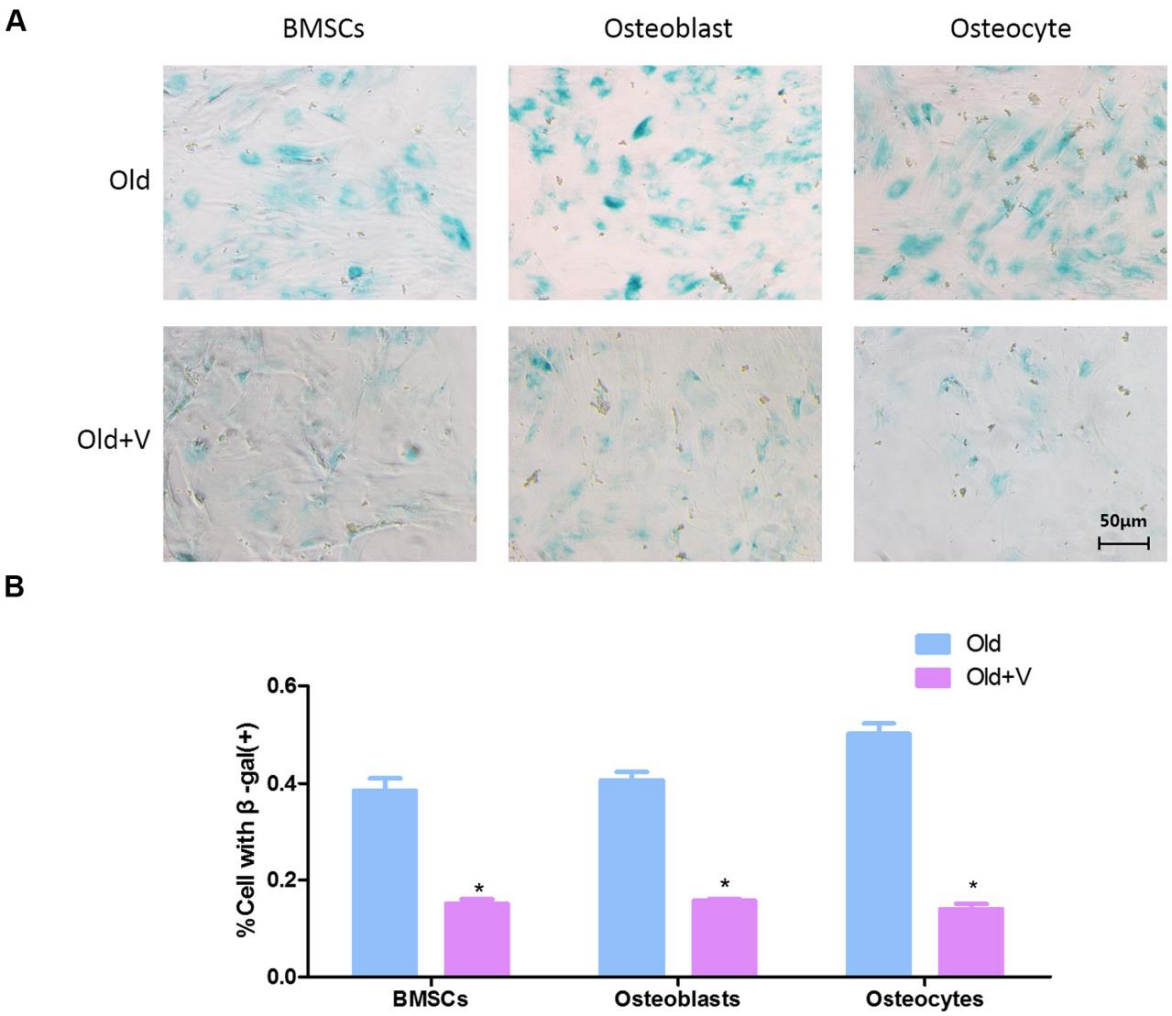
**Low magnitude vibration inhibited osteogenic cells senescence.** (**A**) β-Galactosidase staining of osteogenic cells. Osteogenic cells from aged rats were treated with LMV, thenβ-Galactosidase staining was performed to detect senescent cells. (**B**) Statistical plots of the percentage of positive β-galactosidase staining cells $\left(\bar{x}\pm s,\text{n}=3\right)$; *P < 0.05 vs. old female rats.

Because the previous results proved that there was no significant difference in senescent cells between male and female rats, only female rats were used to verify the effect of low magnitude vibration. As shown in the β-gal staining, there were more β-gal positive osteogenic cells in the old female rats group compared with the young group. And the number of β-gal positive osteogenic cells was decreased significantly after vibration treatment (Figure 7A). Quantitative analysis of β-gal positive osteogenic cells also revealed the same result (Figure 7B).

### Changes in the SIRT1/p53/p21 axis in osteogenic cells after LMV

**Figure 8:**
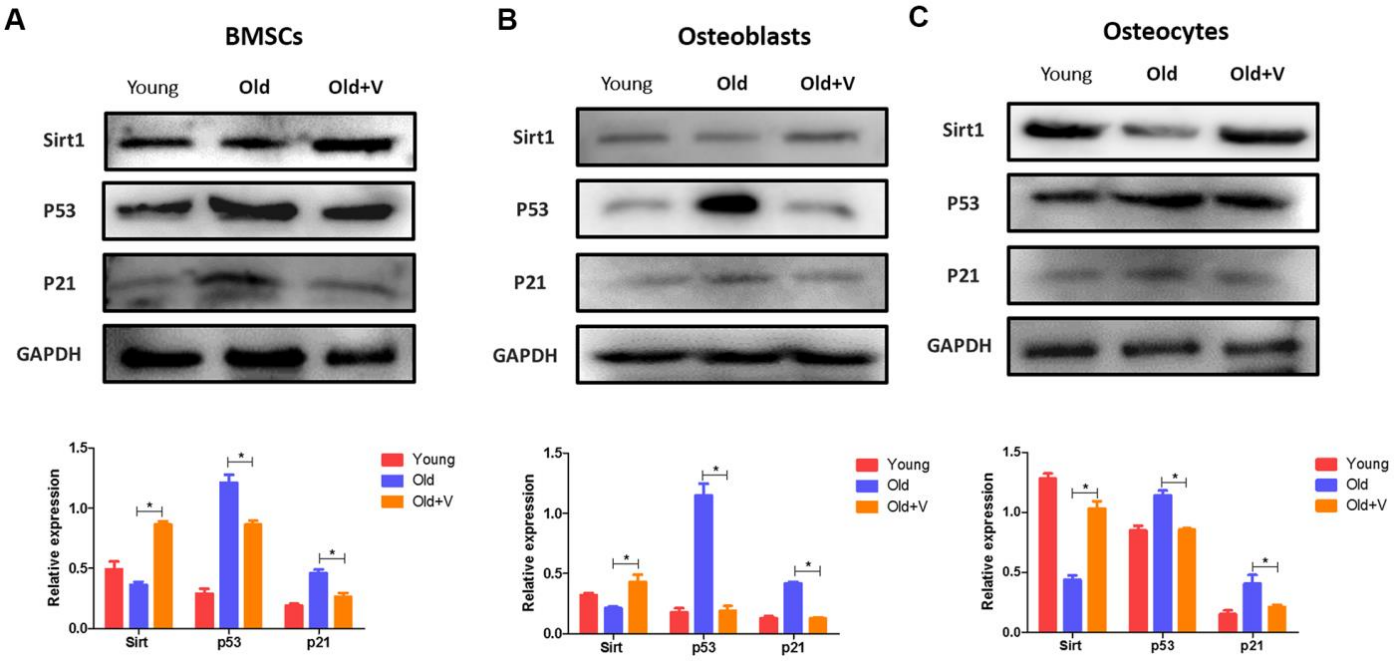
**Changes in the SIRT1/p53/p21 axis in osteogenic cells after LMV.** (**A**) BMSCs were treated with LMV and were harvested to detect the protein levels of SIRT1, p53, and p21 using western blotting $\left(\bar{x}\pm s,\text{n}=3\right)$; (**B**) Osteoblasts were treated with LMV and were harvested to detect the protein levels of SIRT1, p53, and p21 using western blotting $\left(\bar{x}\pm s,\text{n}=3\right)$; (**C**) Osteocytes were treated with LMV and were harvested to detect the protein levels of SIRT1, p53, and p21 using western blotting $\left(\bar{x}\pm s,\text{n}=3\right)$. *P < 0.05 vs. old female rats.

The SIRT1/p53/p21 axis is a typical senescence regulatory pathway. As a well-known anti-senescence protein, SIRT1 was downregulated significantly in senescent osteogenic cells, accompanied by significant upregulation of p53 and p21. However, after LMV treatment, SIRT1 was significantly upregulated with downregulation of p53 and p21 (Figure 8). These data suggested that the SIRT1/p53/p21 pathway was involved in regulating LMV-mediated anti-senescence process of osteogenic cells.

### LMV promotes bone formation of aged rats

**Figure 9:**
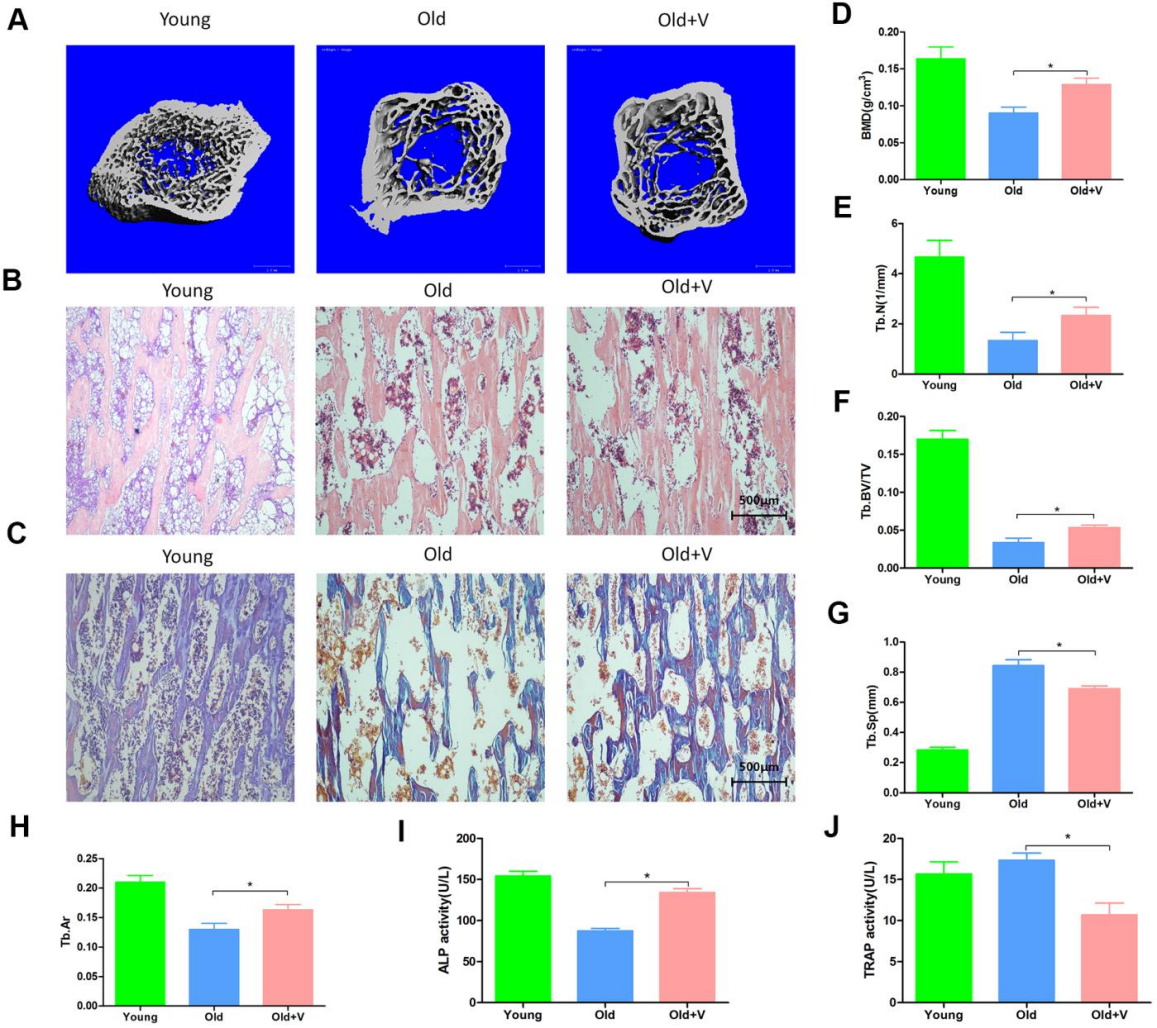
**LMV promoted bone formation of aged rats.** (**A**) uCT of femurs from rats treated with LMV; (**B**) HE staining of femurs from rats treated with LMV; (**C**) Masson staining of femurs from rats treated with LMV; (**D**–**G**) Morphometric parameters of femurs obtained by uCT analysis, including BMD, Tb. BV/TV, Tb. N, Tb. Sp $\left(\bar{x}\pm s,\text{n}=6\right)$; (**H**) Tb. Ar obtained by bone histomorphometric staining analysis $\left(\bar{x}\pm s,\text{n}=6\right)$; (**I**, **J**) Serum ALP activity and TRAP activity detection. *P < 0.05 vs. old female rats.

As shown in Figure 9A, 9D, 9G, the femoral bone density, trabecular volume fraction and trabecular number per unit volume were significantly decreased in aged rats compared with young rats, but partly restored after vibration. Besides, the trabecular separation was significantly decreased in the aged rats after vibration. As shown in Figure 9B, 9C, 9H, after vibration, the trabecular area fraction of femur was significantly increased in aged rats. The analysis of serum biomarkers showed that the ALP activity in the vibration group was increased compared with the static group, while the serum TRAP activity in the vibration group was decreased (Figure 9I, 9J). The above results indicated that aged rats had significant osteoporotic bone structure changes and significant bone loss, and LMV promoted the bone formation of aged rats.

## DISCUSSION

With the increase of age, the functions of various organs and tissues undergo aging changes, especially in bone tissues [20]. Although the abundance of senescent cells in tissues is relatively low, even in tissues from older animals, increased senescent cells are thought to have a profound negative impact on tissue function [21, 22]. Therefore, degenerative and pathological changes are at least partly mediated by the basic biological mechanisms of cellular senescence, and cellular senescence may be a possible link between aging and age-related tissue dysfunction [23]. Although the aging changes of various cell types in the bone microenvironment in mice are confirmed, whether the cells in rat bone microenvironment have similar changes is unknown. In order to verify the senescent osteogenic cells in rat model, we primary cultured osteogenic cells-BMSCs, osteoblasts and osteocytes, and identified the senescence phenotype of osteogenic cells. It was found that compared with young rats, BMSCs, osteoblasts and osteocytes of aged rats all highly expressed aging-related marker p16^INK4a^ and p21, and showed DNA damage, heterochromatin aggregation and increased β-galactosidase activity, which were consistent with several key senescence phenotypes shown in neurons, liver cells, and fat cells [24–26]. Our findings indicated that as rats age, osteogenic cells in the bone microenvironment will become senescent. We also found that β-galactosidase positive osteocytes were significantly higher than that of BMSCs and osteoblasts. It can be seen that osteocytes are the cells with the most obvious senescent changes among the three types of osteogenic cells. Our results are similar to those of Farr et al. They found that about 11% of osteocytes were senescent and secreted more SASP, which had a potential negative impact on neighboring cells in the bone microenvironment [7].

As cell senescence is thought to cause inhibition of cell proliferation [27], we compared the proliferation ability of BMSCs, osteoblasts and osteocytes between aged rats and young rats. Our results showed that with the aging of BMSCs, osteoblasts and osteocytes, the proliferation ability of osteogenic cells were both inhibited, while the proliferation ability of osteocytes declined most obviously, indicating that the more pronounced the changes in aging, the greater the inhibition of cell proliferation capacity. Therefore, the decrease in proliferation activity of osteogenic cells in old rats may be related to the aging changes of osteogenic cells.

Although Farr et al. previously identified the aging of osteoblasts and osteocytes in the bone tissue of old mice, they did not identify the aging changes of BMSCs and did not further study whether BMSCs, osteoblasts and osteocytes occur functional changes with aging changes. In this study, we further determined whether there is a decrease in cellular functional activity during the aging process of BMSCs, osteoblasts and osteocytes. We found that the osteogenic differentiation ability of BMSCs in aged rats decreased and the adipogenic ability was enhanced. The mineralization ability, ALP activity and the expression of bone formation protein of osteoblasts in aged rats was decreased. We also found that osteocytes in aged rats up-regulated the negative regulator of bone formation-sclerosteosis. These results suggested that with aging, the biological functions of BMSCs, osteoblasts and osteocytes declined.

Our research is also of great significance for the treatment of senile osteoporosis. The current osteoporosis medications are mostly used for anti-bone resorption rather than to promote bone formation. They cannot restore the lost bone mass, while inhibit the functional activity of osteoclasts and osteogenic cells [28]. Therefore, it is urgent to develop a new therapy for promoting bone formation and treating osteoporosis. In view of the key role of osteogenic cells in regulating bone formation, targeted elimination of dysfunctional senescent osteogenic cells may be possible to enhance bone formation and prevent age-related bone loss. Previous studies had shown that although the abundance of senescent cells in aging tissues is relatively low, the use of transgenic, cell therapy or pharmacological methods to selectively eliminate senescent cells can prevent tissue dysfunction and a variety of age-related diseases [10, 29, 30]. Therefore, eliminating or reducing the aging osteogenic cells may be a feasible treatment to prevent age-related bone loss. Studies had made meaningful attempts to address this problem. Zhang DY et al. used cortin to eliminate aging BMSCs *in vitro* to promote the proliferation and osteogenic differentiation of BMSCs, and to inhibit adipogenic differentiation of BMSCs [31]. Farr et al. used AP2018 in the INK-ATTAC transgenic mouse model to induce the elimination of p16^Ink4a^-positive senescent cells to increase bone formation, bone mass and bone strength in mice [11]. It is worth noting that although transgenic models can be used to eliminate senescent cells and promote bone formation, transgenic methods are difficult to be achieved in humans. Therefore, new technologies should be developed to target senescent osteogenic cells in bone tissues. Yu XQ et al. found that LMV can effectively promote the osteogenic differentiation of BMSCs in aged rats, and improve bone structure and bone biomechanical properties [17]. Therefore, we explore the anti-aging effect of LMV on the bone from aged rats. SA-β-Gal was a method which many studies had used to verify the senescence reversal [32–34]. In our study, we used SA-β-Gal to verify reversal effect of LMV on osteogenic cell senescence. We found the LMV can inhibit the senescence of osteogenic cells. Our results also proved that LMV can restore the impaired bone formation of aged rats. These results suggested that the inhibition of osteogenic cell senescence by LMV was a valuable treatment to prevent or delay osteoporosis. To the best of our known, this was the first study that revealed the inhibitory role of LMV on cell senescence.

We also explore the mechanism of LMV-mediated inhibition of osteogenic cell senescence. Anti-aging protein Sirt1 is closely associated with the occurrence of senescence [35]. In this research, the protein expression of Sirt1 in osteogenic cells from aged rats was significantly downregulated. Meanwhile, Sirt1 can inhibit the downstream p53, thus inhibiting the expression of p21 to inhibit senescence [36]. In this study, p53 and p21 were notably decreased as well, suggesting that the Sirt1/p53/p21 signaling pathway was prominently functioned in aged rats. Because of the key role of Sirt1/p53/p21 signaling pathway in regulating senescence, the cell senescence can be ameliorated by restoring Sirt1 protein level and decreasing p53 and p21 protein level [37]. The previous results had suggested that Sirt1 could be restored by the mechanical stimuli. Thirupathi A et al. found that strength training and aerobic exercise could significantly increase the Sirt1 to cause a decrease of body fat percentage and adiposity index [38]. Another study reported exercise training could enhance the Sirt1 expression to improve cardiomyocyte survival [39]. Also, study showed that long-term exercise training enhanced the expression levels of Sirt1 and provided cardio-protection against aging [40]. Thus, LMV may also exert anti-aging effects through Sirt1. In the present study, the protein level of Sirt1 was significantly restored with LMV treatment. More importantly, LMV not only restore the Sirt1, but also inhibit the p53 and p21 to attenuate osteogenic cell senescence, which supported the view that LMV might inhibit osteogenic cell senescence at least partly through Sirt1/p53/p21 axis.

## CONCLUSIONS

With aging, the osteogenic cells in the bone microenvironment exhibit aging changes and the decline in proliferation and functional activities. The osteocytes have the most obvious aging changes. However, the LMV can inhibit the senescence of osteogenic cells partly through the Sirt1/p53/p21 axis, thus promoting bone formation of aged rats. Therefore, the inhibition of osteogenic cell senescence by LMV, is a valuable treatment to prevent or delay osteoporosis.
